# Representation of Insured Persons on Insurance Committees

**Published:** 1913-05-31

**Authors:** 


### NATIONAL INSURANCE ACT DEPARTMENT.

### LV.—Representation of Insured Persons on Insurance Committees.

Very little attention has yet been given in the public press, apart from journals specially devoted to the consideration of Insurance subjects, to the forthcoming election of representatives of insured persons to serve on Insurance Committees. The matter is, however, of great importance to all insured persons, as their interests in the National Insurance Scheme should be voiced by their own representatives on the Insurance Committees, and they should, in consequence, see that the powers conferred upon their own societies are duly exercised. The method of election is admittedly regarded as experimental.

### The Provisions of the Act.

It is necessary in the first place to explain that the provisions of the National Insurance Act (section 59, (2) *a*) require that "three-fifths of the members of each Insurance Committee shall be appointed in accordance with regulations of the Insurance Commissioners, so as to secure representation of the insured persons resident in the County or County Borough, who are members of approved societies, and who are deposit contributors, in proportion, as nearly as may be, to their respective numbers." There is also the proviso to the same sub-section that the regulations of the Insurance Commissioners shall confer on the approved societies the power of appointing the representatives of the members resident in each County or County Borough.

Taking the sub-section and its proviso together it is apparently held by the Insurance Commissioners that, each society, which has members resident in any given County or County Borough, is entitled to representation on the Insurance Committee for that area as nearly as may be in the proportion which the number of its members bears to the total number of insured persons resident in the area. This is probably the intention of the Act, but the sub-section quoted above is by no means clearly expressed, for the proportion alluded to is that existing between insured persons who are members of approved societies and those who are deposit contributors respectively, and there is no direct reference to proportionate representation as between different approved societies. This point should receive attention when the Act is brought under review. The representation of members of approved societies in proportion to their numerical strength within each Committee area is, however, a rational proposition, and if carried out effectively should secure the proper consideration of the members' interests.

### The Forthcoming Election.

It must be mentioned that when the Act came into operation on July 15 last it was essential that Insurance Committees should be in existence, as certain of the duties of the Committees devolved upon them immediately—*e.g.* the provision of sanatorium benefit. Seeing then that there were no means available at the time for determining the likely number of members of individual societies, the Insurance Commissioners exercised their powers of making temporary appointments of representatives of insured persons. These temporary appointments will expire on July 15 next, this being the last day allowed under the order constituting the existing Committees. Hence the necessity for the present election, which is the first of its kind, to refill the appointments falling vacant.

The regulations governing the method of election are at first sight exceedingly complex and difficult to understand, and show most striking evidence of the ingenuity that has characterised all the regulations of the Commissioners. The explanatory circular is little better in this regard, but on careful reading the general principles of the method become apparent, and the officials of the approved societies should have little difficulty, if any, in carrying out their part of the process. It is fortunate, perhaps, that the actual detail work will devolve upon a single returning officer appointed by the Commissioners, who, with his assistants, will be able to master the intricacies entailed in the counting of votes.

### Constitution of Committees and Unit of Representation.

Each Insurance Committee has now completed a return to the Commissioners showing the number of insured persons in its area belonging to different societies. The numbers of insured persons shown in these returns are to be the basis in determining the value of the votes of the respective societies.

The Insurance Committees vary in size according to the requirements of the area for which they are constituted, the maximum being eighty and the minimum forty. In consequence the number of representatives of insured persons varies according to the district. The number of representatives to be elected are set forth in the first schedule to the regulations. Committees of eighty members have forty-eight representatives of insured persons. There are only six Committees with the maximum number, these being those for the Counties of Essex, Kent, Lancashire, London, Middlesex, and Yorkshire (West Riding). The County Committees with thirty-six representatives of insured persons are nineteen in number, and the remaining twenty-four County Committees have twenty-four such representatives each. The Committees formed for County Boroughs have in no case more than thirty-six representatives of insured persons. There are eleven with thirty-six representatives each, including Birmingham, Bristol, Leeds, Liverpool, Manchester, and Sheffield, while the remaining sixty Committees have twenty-four representatives each.

The total number of insured persons in the area divided by the number of the representatives to be appointed is termed the unit of representation.

Thus, if there be 48,000 insured persons in a given area, and the number of representatives to be elected is twenty-four, then for that area the unit of representation is 2,000. No account is to be taken of fractions in this or in any of the subsequent operations in which division is involved.

### Appointment of Members by Large Societies.

If any society has more members residing in any given area than the unit of representation for that area, then for each complete unit it has the right to appoint a representative on the Committee, and is debarred from any further share in the election of the remaining representatives. Thus, the societies with the largest membership are definitely secured representation on the Committee without the possible chance of miscarriage which might result if they were forced to go to election. This is sound in principle, but in view of the fact that societies which have not the requisite unit of representation are allowed to nominate two members and, on election from such nominees, are allowed to express their choice in rotation, the appointment without election may press rather hardly on societies whose membership in any given area is greater than a single unit, but not quite sufficient to entitle it to two seats on the Committee. In a similar manner the deposit contributors are to be represented on the basis of the number of complete units of which they consist, except that in their case one representative is to be allowed them whether the first unit is completed or not. It is to be feared that deposit contributors have no associations of their own; but if in any district such an association be formed, and approval thereof is granted by the Insurance Commissioners, then that association will have the privilege of appointing its own representative or representatives on the Committee. If this right be not exercised the appointment or appointments to represent deposit contributors will be made by the members of the Committee other than those representing insured persons.

### Novel Method of Election.

If an approved society has not sufficient members in an area to entitle it to a direct appointment on the unit basis, it will have the right to nominate two candidates for election. If two such candidates are nominated one must be a woman. The nominations have to be made in prescribed form, and it is important that all who have the right of nomination, and wish to use it, should follow the directions of the regulations precisely, otherwise it will be invalid. Should the number of valid nominations exceed the number of vacancies to be filled by election, a poll will be required. In these circumstances, which will most probably be the general case, the returning officer will post to each approved society which has members in the area a ballot form on which the names and addresses of the candidates, and the names of the societies by which they have been nominated, will be set out. These ballot forms will be completed by writing the figure " 1 " against the name of the candidate first chosen, and the figures 2, 3, 4, etc., in sequence against the names of those next chosen. There is no necessity to fill in any number except " 1 " unless it be desired so to do. The ballot forms must, in similar manner to the nomination forms, be authenticated by the signature of the secretary and two other members of the committee of management of the approved society. Each ballot paper will contain a note of the value of the votes recorded thereon. This will be the numerical strength of the membership of the society within the particular Committee area, and in the first instance each vote will have this value. The full details of the method of counting are too complex to describe here, but the principle is briefly this. In the first place the total value of the valid votes will be ascertained, and this number divided by the number of vacancies to be filled by election will be termed the quota. The ballot papers will first be sorted according to the first choice recorded thereon. Those candidates who are credited with votes of total value equal to, or greater than, the appropriate quota will be declared elected. The ballot papers credited to those with more than a full quota will then be examined and re-sorted according to the second choice expressed. These votes will not, however, carry their full original value, but only the proportion of their original value as will give in total the surplus on the first count. The votes will then be transferred to the credit of the candidate of second choice, at the then existing value, and should the total of the original votes and of those transferred amount to the quota the candidate so receiving them will be declared elected. So the method proceeds until by a process of exhaustion the whole of the vacancies are filled.

The description of the process is still further complicated in the regulations, because an endeavour has been made to secure a minimum representation on each Committee of at least two female members.

It remains to be pointed out that insured persons individually have no vote. The nomination and election of representatives is reserved exclusively to the principal officials of the society to which he belongs. If, therefore, an insured person wishes to be elected on an Insurance Committee, or if he wishes to have any hearing in the matter of the choice of his representative, it is to the officials of his society that he must express his views.

It should be noted that the latest time for receiving nominations is 12 noon on Monday, June 9. Ballot papers, it is expected, will be issued on June 20, and if this be so the latest date for the return will be 12 noon on Friday, June 27.

